# ESPO AND ACADEMY FOR GERONTOLOGY IN HIGHER EDUCATION SECTION SYMPOSIUM: INTERGENERATIONAL ENGAGEMENT IN HIGHER EDUCATION: SUCCESSES, SETBACKS, AND BEST PRACTICES

**DOI:** 10.1093/geroni/igae098.0181

**Published:** 2024-12-31

**Authors:** Abigail Stephan, Janelle Fassi, Carson De Fries

**Affiliations:** Chair; Co-Chair; Discussant

## Abstract

Connecting generations through experiential learning opportunities in higher education provides promise for mutually beneficial outcomes. While the theoretical underpinnings (i.e., the “why”) are clear, the practical application (i.e., the “how”) of bringing undergraduate students and older adults together can be more messy. This session focuses on lessons learned, strategies for success, and setbacks to be aware of, with the goal of enhancing our fortitude as educators who value and strive to incorporate impactful intergenerational experiences into the student experience. The first presentation explores the perspectives of students and older adult participants engaged in an intergenerational team-based learning course for first-year undergraduate students, framed by Age-Friendly University principles. The second presentation shares lessons learned from a new service-learning model in which students assessed the transportation needs of older adults engaged with the university-affiliated Osher Lifelong Learning Institute. The third presentation examines the experience of undergraduate students engaged in hands-on gerontology research and highlights takeaways based on students’ intergenerational connections with older adult participants. The final presentation sheds light on sustainable program development through describing their creation of a checklist that aids in intergenerational technology program implementation within higher education. In addition to drawing on the perspectives of scholars across the academic career-span, this symposium session highlights the many contexts within higher education in which intergenerational connections can be facilitated. Aligning with the Annual Scientific Meeting 2024 theme, these presentations demonstrate fortitude in refining processes to ensure we are building optimal experiences that connect students and older adults.

